# “Actually, it's pretty much like normal PE”: reconstructing social hierarchies from the perspective of visually impaired students and their teachers in segregated PE

**DOI:** 10.3389/fspor.2025.1582648

**Published:** 2025-11-17

**Authors:** Brigitta Höger, Stefan Meier, Martin Giese

**Affiliations:** 1 Department of Sport and Human Movement Science, University of Vienna, Vienna, Austria; 2 Department of Education, Sport Pedagogy, University of Marburg, Marburg, Germany

**Keywords:** ableism, visual impairment, physical education, social hierarchies, inclusion

## Abstract

**Purpose:**

Blind and visually impaired (BVI) students frequently report negative experiences in inclusive Physical Education (PE), often facing social exclusion. Many transfer to special schools, however, research on social inclusion and exclusion dynamics in segregated PE remains scarce. This study examines how BVI students and their sighted PE teachers navigate ability-related social hierarchies in a segregated school in Austria. The investigation is grounded in the concept of ableism and an intersubjective understanding of inclusion.

**Materials and methods:**

Following Clark's Mosaic Approach, participant-led school tours were conducted along with semi-structured guideline interviews with 19 BVI secondary school students and three sighted PE teachers. Data were analyzed using thematic content analysis.

**Results and conclusion:**

The analysis identified three key social hierarchies in segregated PE: (1) the differentiation between sighted students and BVI students, reinforcing the perceived necessity and benefits of segregated PE from both student and teacher perspectives; (2) the differentiation between visually impaired and blind students based on their level of vision, which is embedded in teaching practices and internalized by students; and (3) the differentiation between students' developmental stages as perceived by teachers vs. students' own self-perception, leading to tensions between necessary instructional adaptations and the risk of infantilization. The results illustrate that while feelings of inclusion can be fostered for BVI students in segregated PE by critically dismantling ableist norms of visual abilities, ableist notions can still persist in nuanced, subtle and implicit ways.

## 1 Introduction

In the context of global social transformation, education systems play a crucial role in addressing the exclusion of marginalized groups (1, 2). Aligned with social justice discourses, educational policies strive to ensure equal opportunities for education and social participation for as many individuals as possible. Disability occupies a central position in these efforts, as highlighted by the *Key Principles for Promoting Quality in Inclusive Education* by the European Agency (3) and the *Convention on the Rights of Persons with Disabilities* (CRPD, 4). This paper contributes to research on how physical education (PE) for blind and visually impaired (BVI) students can foster a more inclusive and equitable society.

Globally, inclusive education is closely associated with the dissolution of segregated institutions, as mandated in Article 24, paragraph 2(a) of the CRPD, which states that “persons with disabilities are not excluded from the general education system on the basis of disability” (4). In Anglo-American contexts, BVI students (without additional disabilities) are predominantly educated in mainstream schools (5). However, this trend is not universal and does not apply to German-speaking countries, where inclusive education is unevenly implemented, and special schools remain prevalent, particularly for students with sensory impairments (6–8). In these regions, scholars have highlighted that the interpretation of the CRPD remains particularly contentious (9, 10). For instance, in Germany, recent data show that over half of students with special educational needs (SEN) in Germany—55.9% overall and 50.37% of BVI students—remain in special schools (11), highlighting the persistence of segregated education (12). In Austria, 34.4% of students with SEN were enrolled in special schools in 2022, while 2.5% of SEN students identified as having a visual impairment (13). No data, however, clarifies how many BVI students attend general vs. special schools.

Against this background, we propose that understanding inclusion merely as a matter of spatial (non-)segregation would be overly simplistic. Instead, we argue that, regardless of the educational setting, BVI students must receive the necessary support to feel included in their PE lessons, an argument also emphasized in recent inclusion research (14, 15). However, previous research has often marginalized their voices by prioritizing the perspectives of non-disabled peers, teachers, or parents (16). This exclusion limits our understanding of the thoughts, feelings, and experiences of disabled students, which are essential for creating supportive educational environments. To bridge this gap, we adopt an intersubjective understanding of inclusion as the experience of feeling acceptance, value, and belonging (17), focusing on how BVI students themselves perceive and experience participation in PE.

Existing research highlights that BVI students frequently report negative experiences in inclusive PE (18). For example, they often face bullying, exclusion, and isolation (19). Moreover, facilities and equipment have been shown to remain shaped by ableist norms that fail to accommodate their needs (20). Sighted peers and teachers often appear indifferent to the needs of BVI students, showing limited willingness to reflect on social dynamics or adapt teaching practices. This reluctance, as noted by Ruin et al. (21), is tied to socially constructed norms of normality and ableist attitudes, further relegating BVI students to lower positions within social hierarchies. While these dynamics have been studied in inclusive PE, research on social inclusion/exclusion in segregated settings is scarce (22). In this regard, investigating segregated PE in German-speaking contexts contributes uniquely to the existing body of knowledge: Unlike inclusion-oriented education systems in many Anglo-American countries, Austria, Germany, and Switzerland maintain comparatively high rates of school segregation for students with disabilities (23). These institutional frameworks offer a distinct lens through which to explore how ableist norms are reproduced, negotiated, or challenged within formally segregated environments. By centering the lived experiences of BVI students, this study adds context-specific insights that have been underrepresented in international PE research so far.

We acknowledge that inclusive PE should remain the ultimate goal. However, as long as segregated schooling systems persist, it is essential to critically examine segregated PE settings as well. While these environments may address students' needs more effectively than poorly implemented inclusive settings, they are not without challenges. Students with similar (dis)abilities are not a homogeneous group, and prioritizing certain needs over others may inadvertently create new forms of exclusion. A broader perspective that considers societal norms shaping inclusion and exclusion is needed.

A cultural understanding of disability, together with the concept of ableism, is well established within Critical Disability Studies and has recently gained prominence in sports pedagogic discourses (24, 25). This perspective rejects an individualistic or medical model that defines disability as an inherent attribute tied to functional limitations (26). Instead, the cultural model examines societal notions of normality. These notions are shaped by a “network of beliefs, processes and practices that produces a particular kind of self and body (the corporeal standard) that is projected as the perfect, species-typical and therefore essential and fully human. Disability then, is cast as a diminished state of being human” (27). This shift of focus highlights societal discourses and norms that define what counts as a “normal” or “able” body and, by extension, what is deemed “deviant” or “disabled.” Such discursive constructions are closely tied to the concept of ableism. Ableism can be understood as “a set of beliefs, processes, and practices that produce, based on one's abilities, a particular kind of understanding of one's self, one's body, and one's relationship with others of one's species, other species, and one's environment, and includes one being judged by others” (28). From this perspective, dis/ability emerges as a social construct—akin to gender or race—produced through the intersubjective and societal attribution, or more commonly the denial, of abilities (29). This process classifies individuals as “able-bodied” or “disabled” and reinforces the “ableist divide” (27). Notions of “normal” and “deviant” abilities are also context-specific, giving rise to nuanced variations of how individuals are classified as “more” or “less able” across different environments. These classifications form the basis for processes of social inclusion and exclusion. The attribution or denial of abilities leads to what has been described as sub-segregation (21, 22): the (re-)production of ability-related social hierarchies that underpin inclusion and exclusion. Examining these hierarchies constitutes the central objective of our study.

To contribute to this body of research and the identified gaps, *we investigate how BVI students and their sighted PE teachers negotiate ability-related social hierarchization in segregated PE as a basis for social in- and exclusion*. Insights from this study aim to improve participation opportunities in segregated PE, which will likely remain a reality in German-speaking education systems in the future, while also contributing to improving experiences of BVI students in inclusive PE and sports settings.

The following sections outline our research context and methodological considerations, followed by a description of participants and ethical considerations, our methods of data collection and analysis as well as a critical reflection of our positionality as researchers. We then present the results of our data analysis and conclude with a discussion of implications for fostering participation and inclusion in PE for BVI students.

## 2 Materials and methods

Our data originates from a larger research project on the participatory development of digital assistive technologies for PE based on the ideas and self-identified needs of BVI students (16, 20). To establish a foundation for these participatory processes, we first sought to better understand the subjective experiences of BVI students and their sighted teachers in segregated PE.

In line with an intersubjective understanding of inclusion as opposed to spatial integration, educational spaces—such as schools, classrooms or gyms—are far from neutral or passive spatial backdrops. Instead, they are social constructs, continuously (re-)shaped through the interactions of people, spaces and objects (30), where learning unfolds as an embodied experience (17). Capturing the situated and embodied knowledges (31, 32) of BVI students in PE requires data collection within the specific environments of interest—in this case, the school spaces where PE occurs. Clark's Mosaic Approach (30, 33) provided a highly suitable methodological framework for this investigation. This multi-method, participatory, and reflexive approach enables the reconstruction of children's and adolescents' embodied experiences within educational institutions. From Clark's extensive range of proposed methods, we deliberately selected those that enabled students and teachers to share their experiences most authentically (30) while minimizing the risk of reinforcing ability-related hierarchies. This consideration was crucial, as certain methods could require varying levels of assistance depending on the degree of visual impairment, potentially creating disparities among participants. Since all students had completed extensive mobility training, enabling them to navigate the school grounds independently regardless of their visual abilities, we conducted participant-led school tours accompanied by semi-structured guideline interviews. Interview questions were tailored to elicit the perspectives of both students and teachers on the topics of interest. Students participated in pairs or small groups, while teachers were interviewed individually. Small group interviews with students were used to mitigate power asymmetries between researcher and participants and to draw on the supportive dynamics of peer interaction. To account for pre-existing peer relationships, students were allowed to choose their group composition, while the interviewer facilitated balanced speaking time and ensured that differing viewpoints were respected. Teachers were interviewed individually to evoke rich reflections of their own teaching practices and avoid collegial pressure (16). In addition to verbal data, we collected field notes and photographs of places and objects that students and/or teachers identified as significant to their PE experiences.

### 2.1 Participants and ethical considerations

The data were collected in a public special school for BVI in Austria. 19 students (12 female, 7 male) aged 14–20, participated in the study. Among them, three were blind, while the remaining 16 were visually impaired. None of them had any additional disabilities. This comparably large sample represented a quite wide spectrum of visual abilities allowing for a nuanced exploration of how varying types and degrees of visual impairment shaped students' embodied experiences, social positioning, and participation in PE contexts.

All blind students had attended the school since primary level, whereas the visually impaired students had transitioned from inclusive settings either at the start of lower secondary school or during it, based on teacher recommendations and their own reports of unmet educational needs in inclusive environments. 17 students had previously attended schools in Austria, while one male student had transferred from an inclusive lower secondary school in Germany and one female student transitioned after attending an inclusive primary school in Syria and an inclusive lower secondary school in Austria. Additionally, their three sighted PE teachers (2 female, 1 male) aged 43–48 participated in the study. All were Austrian and had over ten years of experience teaching PE to BVI students (Table 1).

**Table 1:** Participants.

Pseudonym	Role	Age	Gender	Self-description of vision	Degree of VI
Michael	Student	16	Male	“I just see a bit worse and just have to look twice to see something. Like look at stuff longer to recognize it sometimes. It depends”	Visually impaired
Vanja	Student	18	Female	“Of course, I realize that I'm limited by a lot of things, but I have accomplished a lot in life. Sometimes, I still struggle, but I think everyone does. I wouldn't say that I have coordination problems. I play an instrument, several instruments, numbers are not my friends, but apart from that … yeah”	Blind
Lina	Student	14	Female	“I had an orientation day here, and I saw that the classes are much smaller, in my other school there were more people in one class with different needs. They would have minded visual impairments but also other kinds…”	Visually impaired
Samira	Student	16	Female	“I have come to kindergarden here since I've been four or five. I was at another kindergarden before but wasn't really supervised properly which caused an accident. That's why my mom found this school for me”	Visually impaired
Laura	Student	16	Female	“According to the doctors, I should actually not see anything at all, I was diagnosed with optic hypoplasia. Long name and difficult to memorize. With that illness you usually don't see anything, but I can see like shadow silhouettes”	Blind
Emma	Student	15	Female	“So, I have REP, I can see in the center, but not on the outsides. My glasses don't really help, they make everything a bit sharper, but I don't see much different with them. I cope quite well, it's just small things (fig.), like with reading, small letters, I can't really read those well, or when things are further away. But apart from that it's pretty good”	Visually impaired
Ayse	Student	19	Female	“I have Morbus Stargard, but I don't really know the details, because I don't like to research that. My left eye sees better than the right one, but the right one supports the left one. I think I can recognize and see stuff pretty well. But of course, the details … Sometimes you don't quite pay attention, or you're not focused or tired. It really depends on the situation, too”	Visually impaired
Sarah	Student	16	Female	“So, for me, I have macular degeneration. That's an illness or a visual impairment, where you can't see that well in the center. Also on the edges, but for me, it's mostly in the center where I don't see. There is this spot, where I don't see. I also have diopters, but it's not something that glasses would help with. Because you can't really determine whether it would be better because of the other illness. And yeah, I get along quite well in life. It's like the small things (fig.) that are hard, especially, you realize when you have to manage on your own. And like with reading and stuff, that's not so easy”	Visually impaired
Zahra	Student	14	Female	“So, I can see 15%. I can see everything from afar, but when it's like writing, I really can't. I mean, I can see the colors, like black and white, but I can't recognize the letters”	Visually impaired
Nuri	Student	14	Female	“I can see pretty much everything, but I can't quite recognize what it is from afar. Like it's a person, I can see that, but I can't recognize who it is”	Visually impaired
Kerstin	Student	14	Female	“For me it's different, because my eye percentages are different. (I: And which kinds of things do you recognize well?) I can recognize pretty much everything”	Visually impaired
Luca	Student	15	Male	“I can still recognize and read things when I get really close. And I can recognize faces a bit. (I: And what is it like in PE?) I don't have any issues there, I can play soccer perfectly, I was on a soccer team, but then I wasn't into that anymore. Because it was also a bit difficult. You have to play soccer in winter too, and when it was getting dark, I had a hard time. Then I quit”	Visually impaired
Emir	Student	16	Male	“I am blind in one eye. And the other one is so-so. So, I can still manage, but yeah”	Visually impaired
Noah	Student	16	Male	“So, for me, I can still recognize silhouettes, but not that well anymore. I can recognize bright and dark, but that's it. (I: And in PE?) Kind of difficult”	Visually impaired
Maximilian	Student	20	Male	“So, I have just below 70% visual impairment, couple of percent, I can't really say. […] I mostly see colors. Things get blurry, but I recognize things through colors. […] Doctors told me that it should not get any worse”	Visually impaired
Liam	Student	17	Male	“On my disability card it says 70% visual impairment. I would be able to manage on my own on public transport and so on (laughs), but with my eye disease I have a hard time discerning dark colors. But apart from that … yeah”	Visually impaired
Elena	Student	15	Female	“So, for me, I need darkness, and I can't really see things far away. Like, I can see you, but for example I need writing really close to me […] And yeah, I can actually see everything in this room. The doctors say I have like 2% [remaining vision], but I don't feel like that at all. I feel like I have 10, 11%”	Blind
Sasha	Student	14	Female	“From far away I can't really read well. But when it's like six or five or eight cm away from me, I can read it. But when it's really far away from me, I can't see it at all. And sometimes it depends on the size of the letters. For instance, I sometimes can't read handwriting. But when it's computer writing, I can read it”	Visually impaired
Nikita	Student	14	Male	“So, I have 10% remaining vision, and it's like with Elena. It's easier in the dark, I see better. And uhm, I think I can read letters normally, I just need minimal magnification. But I have a hard time with farther distances, like 1 m. Color recognition, so when I have like green and blue in front of me, I have kind of a hard time discerning those two, but apart from that it's easy for me to recognize colors. Yeah, that's it”	Visually impaired
Susanne	Teacher	48	Female		Sighted
Claudia	Teacher	43	Female		Sighted
Thomas	Teacher	47	Female		Sighted

Before data collection, written informed consent was obtained from all participants. Students indicated that printed consent forms, accessible via text-to-speech applications on their smartphones, would be most convenient. Accordingly, printed forms were provided in age-appropriate language for both students and teachers. Terms of participation were thoroughly discussed, including the right to withdraw from the study at any time. Ample time was given to address questions, and participants were assured that students' responses would not be shared with their teachers and vice versa.

### 2.2 Methods of data collection and analysis

The inquiries began in empty classrooms, gym halls, or locker rooms with a general discussion about participants' PE experiences. These conversations included broad descriptions of lessons, favorite and least favorite activities, and progressed to questions about the most significant spaces, people, and objects shaping their experiences in PE (30). In the main part of the interviews, students and teachers guided us through PE spaces they deemed most relevant, highlighting aspects they liked, disliked, or wished to retain, eliminate, or modify for a more accessible PE in the future. This approach was designed to give participants maximum freedom while providing enough structure to encourage the sharing of their experiences. By combining interviews with an exploration of the physical spaces, participants could more fully convey their embodied experiences of PE compared to interviews conducted in spatially unrelated settings. Importantly, we avoided directly addressing students' impairments or participation barriers to minimize imposing our own internalized ableist assumptions or unnecessary problematization of visual impairments in ways that might not be relevant to them (27).

All interviews and school tours were audio-recorded and transcribed verbatim, using pseudonyms to ensure anonymity. Data were analyzed using thematic analysis (34) in MAXQDA (35). Coding was performed deductively by the first and second author along two categories derived from theoretical concepts (1): distinctions among students based on corporeal standards, reflecting norms that differentiate “normal” from “deviant” bodies (27), and (2) distinctions based on ability expectations, determining who is deemed “able” vs. “less able”/“disabled” (28). Related codes were then grouped into broader themes according to identified patterns. Together with the third author, we reviewed emerging themes for coherence, refining and finalizing them.

### 2.3 Researchers' positionality

It is important to be transparent about how the researchers' positionalities may have influenced data collection, analysis, and interpretation (36). The first author identifies as a white, cisgender, non-disabled woman from an educated middle-class background. She is healthy and physically active, with a professional background in PE and is currently a PhD candidate in Sport Pedagogy. Her academic work focuses on intersectional power relations and discursive constructions of the body in PE. The second author identifies as a white, cisgender, non-disabled man from a more or less middle-class background. He is physically active, with a professional background in PE and Sports Sciences. Currently, he serves as Full Professor of Sport Pedagogy and head of the Institute of Sport Science at his university. His research centers around diversity and inclusion as well as professionalization of PE teachers/PE teacher education. The third author identifies as a white, cisgender, non-disabled man from an educated middle-class background. He is physically active, has a decade of experience as a special education PE teacher for BVI students and currently serves as Full Professor of Sport Pedagogy and Deputy Managing Director of the Institute of Sport Science at his university. His research focuses on inclusion and disability in the context of visual impairment and blindness, Bildung and lived experience, and digitality.

As a research team, we were aware of the asymmetrical power relations embedded in our roles as adult academic researchers working with children and adolescents in a segregated educational setting. These asymmetries were shaped not only by age and institutional position but also by differences in ability, bodily normativity, and professional authority. Our varying disciplinary backgrounds and lived experiences informed how we engaged with the field, the participants, and the data.

To mitigate potential bias and foster critical awareness throughout the research process, we engaged in regular peer reflections during data collection and analysis. These reflections served as valuable moments for mutually questioning our assumptions and interpretations. In addition, our methodological decisions were shaped by a shared concern to avoid reinforcing ability-related hierarchies during the data collection process. Drawing on Clark's Mosaic Approach (30, 33), we carefully developed a methodological repertoire that enabled students to express their embodied experiences authentically, while minimizing the risk of privileging certain sensory or cognitive abilities. This reflexive approach was central to our effort to foreground the perspectives of students and teachers while critically engaging with our own normative assumptions about ability.

## 3 Results

The analysis of data revealed three interrelated themes that became relevant from the students' as well as the teachers' perspective regarding how ability-based social hierarchies are negotiated among BVI students and their PE teachers within segregated PE. These three social hierarchies will now be described individually, followed by an exploration of their relation and interconnectedness in the discussion.

### 3.1 Social hierarchies between students with and without BVI

The first theme emerging from the analysis highlights the social hierarchization between sighted students and BVI students in inclusive and segregated PE, respectively. Both the participating BVI students and their PE teachers conceptualize BVI students as deviating from the norm of sighted students and organize PE into two distinct spheres: inclusive PE and segregated PE. The teachers articulate this division by frequently invoking an “inside-outside logic”, referring to the “inside” of the special school and the “outside” of the inclusive schooling system. The “outside” of inclusive PE is characterized by both groups as imposing unjustified expectations of abilities on BVI students. In contrast, the “inside” of segregated PE is defined by the recognition that such ability expectations for BVI students are unwarranted and unrealistic. For instance, Emma and Ayse report about their past experiences in inclusive PE:

Ayse: “[It was a] disaster. We didn't really have proper PE classes; instead, they mostly had us play games like dodgeball or memory ball. The problem is, if we can't see, we can't catch the ball.”

Emma: “Or what's it called… volleyball. That was all so difficult.”

Ayse: “Everything with balls is just so difficult. For people with disabilities or blind people, it's really hard when we can't see the ball coming and then can't catch it or things like that.” (Student Interview 3:74–76)

PE teachers and BVI students elevate adapted conditions in segregated PE to a necessity and new norm that addresses the individual needs of BVI students. However, their reasoning differs: the BVI students largely perceive segregated PE as an opportunity to engage in similar movement experiences as their sighted peers in inclusive PE, only under adapted conditions (e.g., adjusted learning pace or tailored instructions), allowing them to experience a sense of belonging. Four students, Emma, Zarah, Nuri and Kerstin describe their experiences as follows:

Emma: “Of course, it's different from PE in regular schools. All of us have been to regular schools at some point, and it's obviously different there. But the teachers and students here do pay attention to how we manage things. Here, it's more tailored to us so that we can actually participate.” (Student Interview 3:71)

Nuri: “Well, actually, it's pretty much like normal PE.”

Zahra: “Except they give a lot more instructions.”

Kerstin: “Because some of us are visually impaired and can't see exactly what we're doing, or some are blind, so things have to be explained in more detail.”

Zahra: “And it's also much slower. The teachers speak more slowly and repeat things a few times.”

Nuri: “But other than that, it's all normal, just like in regular classes.” (Student Interview 4:6–10)

From the analysis it becomes evident how BVI students’ feelings of normalcy are closely linked to experiences of social in- and exclusion. The data illustrates how students associate adapted PE not with segregation or deficiency, but with the opportunity to participate on equal footing, though under different conditions. Crucially, this sense of “normalcy” is not only tied to the structure of the lessons but also to broader feelings of social inclusion. The wish to not differ too much from peers in mainstream schools surfaces repeatedly in the students' accounts, revealing how the experience of belonging is negotiated in relation to imagined norms of able-bodied schooling:

Lina: “Even though we're different, we can still enjoy PE.”

Researcher: “What do you mean by that?”

Lina: “Well, because the others—oh my God—like, so we don't feel as excluded from the other schools, the regular schools. As if we are so different.”

Samira: “We're different from the other schools. Like, we're a special school. And so we don't want to be too different from the other schools, even in sports.” (Student Interview 2:32–35)

Conversely to students’ perspectives, the PE teachers justify adaptations in segregated PE based on specific support needs, vulnerabilities, and protective requirements of their BVI students to mitigate potential negative consequences. While they strongly emphasize the necessity to provide BVI students with opportunities of participation, this notion is also accompanied by explicitly lowered expectations of ability and limited developmental potential for BVI students. Certain activities are deemed meaningless and are therefore excluded from PE, while specific sports equipment is avoided altogether to prevent what is considered inevitable failure, embarrassement or injury. For instance, one teacher describes activities such as throwing or gymnastics as follows:

Thomas: “I hardly know anyone who can throw well as a blind person because the movement is so complex. [..] So, when it comes to things where I know from the start that failure or embarrassment in front of others is inevitable, I have to think about whether it's okay to avoid it entirely or if I can find a way around it.” (Teacher Interview 3:12)

Thomas: “And that would be the balance beam, but we've taken it down. A relic. But it's not really necessary because for our students, just flipping over the long bench is entirely sufficient. Really doing gymnastics is unimaginable anyway.” (Teacher Interview 3:61)

Thus, ambivalent meanings are assigned to segregated PE: On the one hand, segregated PE represents an essential environment for BVI students that facilitates participation in movement and sports experiences in which they can perceive themselves as “normal”. On the other hand, segregated PE is conceptualized as a necessary protective space, legitimized by reduced expectations of abilities and development as well as the heightened vulnerability and support needs of BVI students. In this manner, both PE teachers and BVI students simultaneously challenge and maintain the social hierarchy between BVI students and sighted peers.

### 3.2 Social hierarchies between blind and visually impaired students

As the second theme that emerged from our analysis, it became evident that ability-related normative assumptions and expectations regarding visual abilities served as a criterion for differentiating between blind and visually impaired students, pointing to another mode of social hierarchization. Interestingly, PE teachers did not explicitly articulate this distinction in their interviews. However, students casually reproduced it when describing specific tasks assigned by their teachers during PE lessons. This suggests that PE teachers implicitly reinforce this hierarchy through the didactic structuring of movement tasks.

In the context of diving for objects during swimming lessons, Laura described:

Laura: “Yes, of course, it takes longer; you have to go to the bottom, you have to swim all the way down, and the blind students have to feel everything, and also with breathing time, it certainly takes a bit longer. But actually, it is more intended for sighted people. I mean, blind students can do it too; it's just a bit more difficult. And yes, I always used to do it at the end of the lesson when everyone was already getting ready.”

Researcher: “You were curious and wanted to try it out?”

Laura: “So, I did do it.” (Student Interview 2:147–149)

This highlights how VI students with certain abilities—such as the ability to partially see and thus not having to rely on tactile exploration—are constructed as the “norm”, from which blind students deviate. The latter, in turn, challenge these normative expectations, as their abilities make such tasks nearly impossible, significantly more time-consuming, or even risky. Consequently, blind students are positioned as less capable in direct comparison to their partially sighted peers.

Furthermore, students recount that PE teachers frequently assign VI students to pair up with blind students to assist them with movement tasks. These pairings are not fixed but are regularly rotated. For example, Lina explains:

Lina: “Yes, actually, we always switch. It is NEVER always the same person; it keeps changing. Since there are more sighted students, it alternates—one time one person helps, then another, and so on.” (Student Interview 2:53)

On the one hand, this arrangement serves as a common didactic strategy to facilitate participation for blind students. On the other hand, rather than challenging or mitigating social hierarchies among students with different visual abilities, it may actually reinforce them. VI students are implicitly positioned as responsible and capable helpers, whereas blind students are cast as dependent, reliant on assistance, and unable to act independently. Thus, such didactic arrangements create a “helper child logic”, which perpetuates stereotypes of helplessness and inactivity associated with blind students.

Beyond such enabling logics, students with visual impairments also articulate limitations in the selection of certain physical activities, which they attribute to the perceived limited abilities of their blind peers. They describe how certain games can only be played with significant modifications or are excluded from PE altogether due to the lack of visual abilities among their peers and safety concerns. For example, Ayse recounts that ball games are rarely played at all and, when they are, only with specific adaptations and safety measures in place:

Ayse: “So, we don't play many ball games, because it's difficult for disabled and blind students to play with a ball when they can't see it. But we do play with audible balls, just not dodgeball. We can't shoot too hard or not watch where we're throwing, because that could go wrong. You have to be careful.” (Student Interview 3:63)

Similarly, Nuri expresses her wish to play volleyball but finds that this is deemed impossible specifically due to the assumed limitations of blind students and even adaptations such as audible balls are considered insufficient leading to the activity being excluded altogether.

Nuri: “I would really like to play volleyball somehow. But unfortunately, we can't, because some other students have worse vision. And with the balls that make sounds, it also doesn't really work.” (Student Interview 4:178)

In this manner, normative assumptions and expectations regarding visual abilities give rise to forms of sub-segregation within the segregated PE setting. Students are implicitly differentiated according to the degree of their visual impairment. Those with partial sight are positioned at the top of the social hierarchy, as their abilities are perceived as closer to the norm and allow them to act as helpers or participate more fully in certain activities. In contrast, students with lower vision or blindness are positioned at the bottom. Their participation is often framed as difficult or risky and made dependent on assistance or specific adaptations. Rather than mitigating social hierarchies, the segregated setting thus produces new internal distinctions based on perceived ability, reinforcing ableist notions of competence and independence.

### 3.3 Social hierarchies between students according to assumed maturity

The third theme emerging from the analysis relates to social hierarchizations based on the perceived or teacher-attributed social and cognitive developmental levels of BVI students. Teachers often indirectly construct visual impairment as the cause for why BVI children fall behind expectations of age-appropriate developmental levels, by suspecting that they have experienced a particularly sheltered or patronizing upbringing due to their disability and did not receive the necessary support to develop in what is deemed an age-appropriate manner. These notions of what constitutes an “appropriate” developmental stage for a certain age lead, particularly on the part of teachers, to specific expectations regarding students' pace of development, learning speed, and ability levels. As a result, they adapt their teaching practices to cater to their specific needs.

One teacher, Susanne, talks about one of her primary school classes:

Susanne: “So, I got to know these children in September, and then you're standing there in the lesson, and very quickly, you reach a point where you start to question your own abilities because it's just so challenging to teach these four children. Honestly, they all really belong in kindergarten. But of course, you have to assess that first—it takes time. By the end of September, I decided for myself, ‘Okay, I'll take my ideas two years back.’ And from that moment on, I thought, ‘Hello, we don't need to overcomplicate things here.’ Even a simple movement task is already too much. For example, you let the music play, and when it stops, they all lie down on the floor. Even that kind of input is already too much for these children. You know? […] And then, after conversations with colleagues—who thankfully always feel exactly the same way (laughs)—you decide, ‘Alright, back to kindergarten level.’” (Teacher Interview 1:46)

At the same time, teachers seem to draw conclusions from the assumed developmental levels of the students regarding what content might align with their interests and how it should be appropriately delivered in PE. Such assumptions also appear to translate to the older students at secondary level. These older students, however, do not appear to share this assessment in their self-perception. For instance, Vanja and Michael discuss, how they perceive the teacher's approach to teaching PE as infantilizing and describe their PE lessons as childish:

Vanja: “Well, the purpose is to learn that sports can be fun. I think it's good to focus on games in the first few years, but as you get older and you're in high school, I find games a bit childish.” [..]

Michael: “For me, [PE]'s just a way to pass the time, but honestly, it feels like a waste of time. You warm up nicely, okay, gymnastics, great, and then it's either playing ball games or doing silly things on the mat, like standing back-to-back and having to push each other away.”

Vanja: “We also had to crawl through each other's legs—such nonsense.”

Michael: “Yeah, and I think, ‘I enjoyed that in kindergarten or elementary school, but not in high school.’ And I really feel like she criticizes me a lot and keeps picking on me. She totally misjudges me and sees me as someone completely different from who I actually am.” (Student Interview 1:18–32)

In this way, social hierarchization based on (assumed) developmental levels becomes a point of tension between teachers' attempts to provide adapted and differentiated PE, and the students' own perceptions of themselves, their bodies, and their interests. While teachers interpret certain behaviors or forms of participation through the lens of developmental delay, students reject these attributions by presenting themselves as capable, mature, and misrecognized. These tensions reveal how ableist norms of age-appropriate development and physical abilities are perpetuated in pedagogical decisions, contributing to the (re)production of social hierarchies within segregated PE settings.

## 4 Discussion

The analysis has revealed three distinct forms of social hierarchies that emerge in segregated PE among BVI students and their sighted teachers: (1) the differentiation between sighted students and BVI students, which serves to justify the necessity and benefit of segregated PE from the perspectives of both students and teachers; (2) the differentiation between visually impaired vs. blind students based on their degree of visual impairment, which is embedded into teachers' didactic practices and internalized by students; and (3) the differentiation between developmental stages of BVI students as perceived by teachers vs. students' self-perception, which leads to tensions between necessary instructional adaptations and the risk of infantilization.

The three described forms of social hierarchy are shaped by an overarching ableist regime, which not only becomes apparent on the institutional level in the distinction between mainstream and special schools but also operates within social relations inside the special school, constituting the social hierarchy between sighted and BVI students. While ableist norms are, at least in part, clearly named and critically questioned by students and teachers when it comes to the differentiation vis-à-vis the constitutive “outside” of mainstream schooling, they are at the same time reproduced within the segregated PE setting of the special school as the sub-segregation between blind and partially sighted students. Thus, while ableist mechanisms of classification are destabilized on a broader level, they are simultaneously maintained on a smaller scale within the segregated school by both students and teachers.

In the context of the third social hierarchy, a certain shift in the focus of ableist regimes can be observed—from visual ability toward social and cognitive abilities. While ableist attributions related to visual ability are challenged and destabilized by both teachers and students within the first hierarchy, attributions of delayed social and cognitive development appear legitimate and acceptable from the teachers' perspective in the context of segregated PE. The students, however, clearly resist and reject these attributions. What seems to be intended by teachers as a didactic adaptation to the assumed social and cognitive capacities of the students—framed as pedagogical care—ultimately denies the students opportunities for self-directed and autonomous learning based on their own capacities and interests. In this way, the segregated school system itself is once again reinforced through ableist norms and attributions.

Most notably, the findings illustrate that reinforcing or resisting ableist notions of deviance and normality as well as maintaining or dismantling related social hierarchies cannot be reduced to individual attitudes or personal beliefs. Like all members of (Western) society, BVI students and their sighted PE teachers are deeply embedded in ableist structures and have internalized normative ideas of “able” vs. “unable” and “normal” vs. “deviant” bodies (27, 28), which are not always accessible to them through critical self-reflection.

Contrary to the findings of Ruin et al. (21), the sighted PE teachers in this study demonstrated a high degree of critical reflection on ability-related expectations and ableist assumptions of normality. They were strongly committed to providing differentiated and adapted learning opportunities, tailoring instruction to individual needs, and creating inclusive learning environments where BVI students could engage in diverse movement experiences under modified conditions. As a result, BVI students described their experiences in segregated PE as significantly more positive than their previous experiences in inclusive PE. Within the segregated setting under investigation, they reported a sense of belonging and normality, both considered essential for inclusion (14, 15, 17). However, while these teaching practices facilitated participation, they were also shaped by implicit notions of lowered expectations and limited developmental potential for BVI students. As a result, social hierarchies between sighted students and those with BVI may persist, subtly reinforcing existing ableist dynamics.

Moreover, unlike in inclusive PE, BVI students did not report experiences of overt bullying, exclusion, or isolation in segregated PE (19). Nevertheless, PE teachers' didactic arrangements of movement tasks were implicitly structured around the distinction between partially sighted vs. blind students. Partially sighted students were frequently assigned to assist blind students in movement tasks, a practice intended to enhance participation for the latter. However, this arrangement led to sub-segregation (22), subtly reinforcing the ableist notion that people with blindness are inherently more dependent on help and “less able” than their partially sighted peers. Although blind students in this study did not comment on receiving such kind of help, previous research suggests that help in PE—whether instrumental, caring, or consensual—can have complex effects on disabled students. While some perceive it positively, others experience a loss of independence, threats to self-esteem, or restrictions on participation (37). Similarly, unsolicited help has frequently been reported as an issue by blind individuals, who often experience it as patronizing and condescending, as it assumes incompetence on their part (38). At the same time, rejecting such assistance can be challenging, as individuals may fear being perceived as ungrateful or rude (39).

Similarly, notions of infantilization mark another pervasive social dynamic towards disabled people, a dynamic widely reported in PE (40, 41). Infantilization leads individuals with physical or sensory impairments—such as BVI students—to feel as though they are not taken seriously or treated in a manner appropriate for their age (42). In this context, assumptions of diminished visual abilities appear to extend to assumptions of diminished cognitive abilities and social development. More broadly, recent research highlights that infantilization, alongside charity narratives, plays a central role in maintaining social inequalities from the perspective of BVI individuals (43). They are at risk of being relegated to a social space of “eternal childhood,” a phenomenon also described in relation to intellectual disabilities (44). Within this space, individuals often experience restrictions on their autonomy and are subjected to forms of patronization that limit not only their personal interests and choices but also their fundamental rights, including political agency, healthcare decisions, and sexuality (45). As a result, it seems crucial for PE teachers to prioritize their students' self- and co-determination in segregated PE and continuously take into consideration their interests and requirements to ensure they are treated with respect and taken seriously.

## 5 Limitations and strengths

This study has certain limitations and strengths: One limitation of our study is that all participants were recruited from a single school. While BVI students may face similar barriers across different educational settings, some of their experiences may be context specific. Additionally, selecting only one school may limit the diversity of students' backgrounds, such as social status or parental support. Moreover, none of them had any additional sensory, physical or intellectual disabilities, which may limit the transferability of the results.

However, our study stands out for its relatively large and diverse sample, including students with varying degrees and types of visual impairment. This diversity enables us to present nuanced perspectives, a key strength of our research. Furthermore, our sample includes both students who transitioned from inclusive schooling and those who have attended a segregated school throughout, offering valuable insights into different educational trajectories.

Another limitation is the absence of participants younger than 14. In Austria, adolescents can provide independent consent for research participation at this age, and we were unable to obtain parental consent for younger students due to organizational constraints. While they participated in the broader project on the development of digital assistive technologies for PE, their data could not be recorded for research. However, we later involved them in testing prototypes and providing feedback, ensuring their perspectives contributed to the project.

## 6 Conclusion & outlook

This study contributes to the broader discussion on inclusive and segregated education, emphasizing that genuine social inclusion requires more than simply integrating BVI students into existing frameworks. The analysis reveals that social hierarchies in segregated PE for BVI students exist on multiple levels and are not simply a reflection of individual attitudes but are deeply embedded in societal ableist structures. The findings suggest that, under certain conditions, segregated PE can offer positive and inclusive experiences for BVI students. When ableist notions of normality and ability-related expectations are critically challenged within PE teaching practices, BVI students can have genuinely inclusive experiences. However, well-intended didactic strategies, such as assigning partially sighted students as helpers or tailoring tasks to assumed social development levels, can unintentionally reinforce subtle forms of social hierarchization, leading to paternalism and infantilization. These dynamics risk perpetuating existing ableist norms rather than dismantling them.

There is no shortage of pedagogical concepts aimed at making PE—whether inclusive or segregated—more inclusive in design and delivery. From an ableism-critical perspective that understands inclusion as an intersubjective experience (17), however, we propose that the crucial yardstick for creating truly inclusive settings is to provide disabled students with meaningful opportunities to have a voice in determining what inclusivity actually means. For an extended period of time, BVI students' experiences in PE have largely been represented through the perspectives of sighted teachers, parents, or peers—those “who have not lived nor embodied disability” (32). Such approaches risk reinforcing ableist assumptions of normality and obscure the subjective meanings BVI students attribute to their experiences (14, 27, 28). As a result, research has often sidelined their perspectives, despite the fact that these should be central to any meaningful understanding of inclusion in PE (46, 47).

In light of this, we propose for future research to shift from “researching on to researching with” young people (48). Participatory and co-creative approaches have recently gained momentum as a means to develop more inclusive, empowering, and justice-oriented research and practice (49, 50). They offer potential not only to amplify the voices of disabled students, but also to challenge the power asymmetries embedded in conventional research and teaching practices (51, 52). Similarly, co-creation and co-production frameworks are increasingly used to collaboratively design curricula and didactic recommendations that reflect students' lived realities in PE (53). For instance, Arroyo-Rojas and Hodges (54) argue that systems of peer-support in PE should be developed jointly with students to avoid reinforcing social hierarchies or unintentionally reproducing paternalistic or infantilizing dynamics.

Future research should therefore place greater emphasis on participatory, student-centered methodologies that foreground the intersubjective experiences of acceptance, belonging and value (17). This shift not only aligns with the principles of the UN Convention on the Rights of Persons with Disabilities (4), but also holds potential to reimagine PE as a more inclusive and equitable space for all.

## Data Availability

The raw data supporting the conclusions of this article will be made available by the authors, without undue reservation.

## References

[1] Ainscow M. Struggles for Equity in Education. London: Routledge (2015).

[2] Michelli NM Jacobowitz TJ Campo S Jahnsen D. Education for Social Justice: The Meaning of Justice and Current Research. New York: Routledge (2023).

[3] European Agency for Development in Special Needs Education. Key Principles for Promoting Quality in Inclusive Education—Recommendations for Policy Makers. Brussels: European Agency for Development in Special Needs Education (2009). Available online at: https://www.european-agency.org/sites/default/files/key-principles-for-promoting-quality-in-inclusive-education_key-principles-EN.pdf (Accessed November 10, 2025).

[4] United Nations. Convention on the Rights of Persons with Disabilities. New York, NY: United Nations. (2006). Available online at: https://www.un.org/disabilities/documents/convention/convoptprot-e.pdf

[5] McLinden M Douglas G Cobb R Hewett R Ravenscroft J. Access to learning’ and “learning to access”: analysing the distinctive role of specialist teachers of children and young people with vision impairments in facilitating curriculum access through an ecological systems theory. Br J Vis Impairment. (2016) 34(2):177–95. 10.1177/0264619616643180

[6] Giese M. Subjektive Konstruktionen von Teilhabebarrieren im inklusiven Sportunterricht von blinden und sehbeeinträchtigen SchülerInnen. ZSF. (2021) 9(2):6–23. 10.5771/2196-5218-2021-2-6

[7] Hardman K. The situation of physical education in schools: a European perspective. Hum Mov. (2008) 9(1):5–18. 10.2478/v10038-008-0001-z

[8] Lanners R. Integration vor Separation im Spiegel unserer Statistik. SZH. (2024) 30(09):2–12. 10.57161/z2024-09-01

[9] Ahrbeck B Badar J Kauffman J Felder M Schneiders K. Fachbeitrag: Full inclusion? Totale Inklusion? Fakten und Überlegungen zur Situation in Deutschland und den USA. Vierteljahresschrift für Heilpädagogik und Ihre Nachbargebiete. (2018) 87(3):218. 10.2378/vhn2018.art23d

[10] United Nations. General Comment No. 4 on Article 24—the Right to Inclusive Education. New York, United Nations (2016). Available online at: https://www.ohchr.org/en/documents/general-comments-and-recommendations/general-comment-no-4-article-24-right-inclusive (Accessed November 10, 2025).

[11] Kultusministerkonferenz [Standing Conference of the Ministers of Education and Cultural Affairs]. Sonderpädagogische Förderung in Schulen 2013 bis 2022. Berlin: Kultusministerkonferenz (2024). Available online at: https://www.kmk.org/fileadmin/Dateien/pdf/Statistik/Dokumentationen/Dok_240_SoPae_2022.pdf (Accessed November 10, 2025).

[12] Maher AJ Thomson A Parkinson S Hunt S Burrows A. Learning about “inclusive’ pedagogies through a special school placement. Phys Educ Sport Pedagogy. (2022) 27(3):261–75. 10.1080/17408989.2021.1873933

[13] Gasteiger-Klicpera B Buchner T Frank E Grubich R Hawelka V Hecht P Evaluierung der Vergabepraxis des sonderpädagogischen Förderbedarfs (SPF) in Österreich. Abschlussbericht September 2023. Vienna: Austrian Ministry of Education (2023). Available online at: https://www.bmbwf.gv.at/Themen/schule/bef/sb/spf_eval.html (Accessed November 10, 2025).

[14] Haegele JA. Inclusion illusion: questioning the inclusiveness of integrated physical education: 2019 national association for kinesiology in higher education Hally Beth Poindexter young scholar address. Quest. (2019) 71(4):387–97. 10.1080/00336297.2019.1602547

[15] Maher AJ Haegele JA. Beyond spatial materiality, towards inter- and intra-subjectivity: conceptualizing exclusion in education as internalized ableism and psycho-emotional disablement. Br J Sociol Educ. (2024) 45(4):531–46. 10.1080/01425692.2024.2334272

[16] Höger B Meier S. Exploring marginalized perspectives: participatory research in physical education with students with blindness and visual impairment. J Teach Phys Educ. (2025) 44(4):802–13. 10.1123/jtpe.2023-0325

[17] Haegele JA Maher AJ. Toward a conceptual understanding of inclusion as intersubjective experiences. Educ Res. (2023) 52(6):385–93. 10.3102/0013189X231176287

[18] Giese M Grenier M. “… it’s so funny to just throw off the blind girl” subjective experiences of barriers in physical education with visually impaired students—an emancipatory bad practice approach. Front Sports Act Living. (2025) 7:1515458. 10.3389/fspor.2025.1515458 40104528 PMC11914114

[19] Ball L Lieberman L Haibach-Beach P Perreault M Tirone K. Bullying in physical education of children and youth with visual impairments: a systematic review. Br J Vis Impair. (2022) 40(3):513–29. 10.1177/02646196211009927

[20] Meier S Höger B Giese M. “If only balls could talk …”: barriers and opportunities to participation for students with blindness and visual impairment in specialized PE. Front Sports Act Living. (2023) 5:1286909. 10.3389/fspor.2023.1286909 38162696 PMC10754968

[21] Ruin S Giese M Haegele JA. Fear or freedom? Visually impaired students’ ambivalent perspectives on physical education. Br J Vis Impair. (2021) 39(1):20–30. 10.1177/0264619620961813

[22] Giese M Ruin S Baumgärtner J Haegele JA. “… and after that came me”. Subjective constructions of social hierarchy in physical education classes among youth with visual impairments in Germany. Int J Environ Res Public Health. (2021) 18(20):10946. 10.3390/ijerph182010946 34682691 PMC8535199

[23] European Agency for Special Needs and Inclusive Education. European Agency Statistics on Inclusive Education: 2021/2022 School Year Dataset Cross-Country Report. Brussels: European Agency for Development in Special Needs Education (2024). Available online at: https://www.european-agency.org/resources/publications/EASIE-2021-2022-cross-country-report (Accessed November 10, 2025).

[24] Giese M Ruin S. Forgotten bodies—an examination of physical education from the perspective of ableism. Sport Soc. (2018) 21(1):152–65. 10.1080/17430437.2016.1225857

[25] Giese M Haegele JA Maher AJ. The ableist underpinning of normative motor assessments in adapted physical education. J Teach Phys Educ. (2024) 43(1):72–8. 10.1123/jtpe.2022-0239

[26] Waldschmidt A. Disability–culture–society: strengths and weaknesses of a cultural model of dis/ability. Alter. (2018) 12(2):65–78. 10.1016/j.alter.2018.04.003

[27] Campbell FK. Contours of Ableism: The Production of Disability and Abledness. Basingstoke: Palgrave Macmillan (2009).

[28] Wolbring G. The politics of ableism. Development. (2008) 51(2):252–8. 10.1057/dev.2008.17

[29] Giese M Buchner T. Ableism als sensibilisierenden Folie zur (Selbst-)Reflexion sportunterrichtlicher Angebote. Sportunterricht. (2019) 68(4):153–7.

[30] Clark A. Childhoods in Context. Bristol: Bristol University Press (2013).

[31] Haraway D. Situated knowledges: the science question in feminism and the privilege of partial perspective. Fem Stud. (1988) 14(3):575. 10.2307/3178066

[32] Maher AJ Haegele JA. Disabled children and young people in sport, physical activity and physical education. Sport Educ Soc. (2022) 27(2):129–33. 10.1080/13573322.2021.1967119

[33] Clark A. Breaking methodological boundaries? Exploring visual, participatory methods with adults and young children. Eur Early Child Educ Res J. (2011) 19(3):321–30. 10.1080/1350293X.2011.597964

[34] Braun V Clarke V. Thematic analysis. In: Cooper H Camic PM Long DL Panter AT Rindskopf D Sher KJ, editors. APA Handbook of Research Methods in Psychology Volume 2: Research Designs: Quantitative, Qualitative, Neuropsychological, and Biological. Washington, DC: American Psychological Association (2012). p. 57–71.

[35] MAXQDA. Software for Qualitative Data Analysis. Berlin: VERBI Software (2024).

[36] Goodwin D. Qualitative inquiry in adapted physical education. In: Haegele JA Hodge SR Shapiro DR, editors. Routledge Handbook of Adapted Physical Education. 1st ed. New York: Routledge (2020). p. 163–82.

[37] Goodwin DL. The meaning of help in PE: perceptions of students with physical disabilities. Adapt Phys Activ Q. (2001) 18(3):289–303. 10.1123/apaq.18.3.289

[38] Calder-Dawe O Witten K Carroll P. Being the body in question: young people’s accounts of everyday ableism, visibility and disability. Disabil Soc. (2020) 35(1):132–55. 10.1080/09687599.2019.1621742

[39] Wang K Silverman A Gwinn JD Dovidio JF. Independent or ungrateful? Consequences of confronting patronizing help for people with disabilities. Group Process Intergroup Relat. (2015) 18(4):489–503. 10.1177/1368430214550345

[40] Holland K Haegele JA Zhu X Bobzien J. “They’re either going to find ways to include you or they’re just kind of not”: experiences of students with orthopedic impairments in integrated physical education. Adapt Phys Activ Q. (2022) 39(3):321–40. 10.1123/apaq.2021-0184 35287113

[41] Ruin S Haegele JA Giese M Baumgärtner J. Barriers and challenges for visually impaired students in PE—an interview study with students in Austria, Germany, and the USA. Int J Environ Res Public Health. (2023) 20(22):7081. 10.3390/ijerph20227081 37998312 PMC10671423

[42] Pérez-Garín D Recio P Magallares A Molero F García-Ael C. Perceived discrimination and emotional reactions in people with different types of disabilities: a qualitative approach. Span J Psychol. (2018) 21:E12. 10.1017/sjp.2018.13 29759090

[43] Hernandez Padilla ML Arias Valencia SA. “The way they treat US reflects how they see US”: aspects contributing to social inequities in people with visual disability, a qualitative study. Br J Vis Impair. (2024) 43(3):02646196241265616. 10.1177/02646196241265616

[44] Loftis S. The metanarrative of autism. Eternal childhood and the failure of cure. In: Bolt D, editor. Metanarratives of Disability: Culture, Assumed Authority, and the Normative Social Order. London: Routledge, Taylor & Francis Group (2021). p. 94–105.

[45] Oviedo-Cáceres MDP Arias-Pineda KN Yepes-Camacho MDR Montoya Falla P Guisasola Valencia L. “My disability does not measure me: it is a way of living and being in the world” meanings of social inclusion from the perspective of people with visual impairment. Br J Vis Impair. (2023) 41(1):181–94. 10.1177/02646196211036403

[46] Spencer-Cavaliere N Watkinson EJ. Inclusion understood from the perspectives of children with disability. Adapt Phys Activ Q. (2010) 27(4):275–93. 10.1123/apaq.27.4.275 20956835

[47] Meier S Reuker S. Fachdidaktische Perspektiven zum Umgang mit Heterogenität im inklusiven Sportunterricht—ein kritisch-konstruktiver Überblick. ZSF. (2022) 10(1):76–99. 10.5771/2196-5218-2022-1-76

[48] Fitzgerald H Stride A Enright E. Messy methods: making sense of participatory research with young people in PE and sport. Eur Phy Educ Rev. (2021) 27(3):421–35. 10.1177/1356336X20953462

[49] Clish M Enright E Sperka L Tweedy S. Engaging young people with disabilities in research about their experiences of physical education and sport: a scoping review of methodologies and methods. Eur Phy Educ Rev. (2022) 29(3):1356336X2211415. 10.1177/1356336X221141598

[50] Cammarota J Fine M, editors. Revolutionizing Education: Youth Participatory Action Research in Motion. London: Routledge (2008).

[51] Fitzgerald H Jobling A Kirk D. Listening to the “voices” of students with severe learning difficulties through a task-based approach to research and learning in physical education. Support Learn. (2003) 18(3):123–9. 10.1111/1467-9604.00294

[52] Coates J Vickerman P. Empowering children with special educational needs to speak up: experiences of inclusive physical education. Disabil Rehabil. (2010) 32(18):1517–26. 10.3109/09638288.2010.497037 20568986

[53] Huynh TN Haegele J James ME Arbour-Nicitopoulos KP. Inclusive physical activity practices for disabled children and adolescents. In: García-Hermoso A, editor. Promotion of Physical Activity and Health in the School Setting. Cham: Springer Nature (2024). p. 359–83. 10.1007/978-3-031-65595-1_16

[54] Arroyo-Rojas F Hodge SR. Phenomenological research involving students with disabilities in physical education: a systematic literature review. Quest. (2024) 76(2):135–53. 10.1080/00336297.2023.2206579

